# Comparison of definite chemoradiation therapy with carboplatin/paclitaxel or cisplatin/5-fluoruracil in patients with squamous cell carcinoma of the esophagus

**DOI:** 10.1186/s13014-018-1085-z

**Published:** 2018-08-02

**Authors:** Stefan Münch, Steffi U. Pigorsch, Michal Devečka, Hendrik Dapper, Wilko Weichert, Helmut Friess, Rickmer Braren, Stephanie E. Combs, Daniel Habermehl

**Affiliations:** 1 Department of Radiation Oncology, Klinikum rechts der Isar, Technical University Munich, Ismaninger Str. 22, D-81675 Munich, Germany; 2 German Cancer Consortium (DKTK) Partner Site Munich, Munich, Germany; 3 Institute of Pathology, Klinikum rechts der Isar, Technical University Munich, Ismaninger Str. 22, D-81675 Munich, Germany; 4 Department of Surgery, Klinikum rechts der Isar, Technical University Munich, Ismaninger Str. 22, D-81675 Munich, Germany; 5 Institute of Radiology, Klinikum rechts der Isar, Technical University Munich, Ismaninger Str. 22, D-81675 Munich, Germany; 6 Institute of Innovative Radiotherapy (iRT), Helmholtz Zentrum München, Ingolstädter Landstraße 1, D-85764 Munich, Germany

**Keywords:** Squamous cell carcinoma of the esophagus, Definite chemoradiation, Cisplatin/5-fluoruracil, Carboplatin/paclitaxel

## Abstract

**Background:**

While neoadjuvant chemoradiation therapy (nCRT) with subsequent surgery is the treatment of choice for patients with locally advanced or node-positive squamous cell carcinoma of the esophagus (SCC) suitable for surgery, patients who are unsuitable for surgery or who refuse surgery should be treated with definite chemoradiation therapy (dCRT). Purpose of this study was to compare toxicity and oncologic outcome of dCRT with either cisplatin and 5-fluoruracil (CDDP/5FU) or carboplatin and paclitaxel (Carb/TAX) in patients with SCC.

**Methods:**

Twenty-two patients who received dCRT with carboplatin (AUC2, weekly) and paclitaxel (50 mg per square meter of body-surface area, weekly) were retrospectively compared to 25 patients who were scheduled for dCRT with cisplatin (20 mg/m^2^/d) and 5-fluoruracil (500 mg/m^2^/d) on day 1–5 and day 29–33. For the per-protocol (PP) analysis, PP treatment was defined as complete radiation therapy with at least 54Gy and at least three complete cycles of Carb/TAX or complete radiation therapy with at least 54Gy and at least one complete cycle of CDDP/5FU. While patients who were scheduled for dCRT with Carb/TAX received a significantly higher total radiation dose (median dose 59.4Gy vs. 54Gy, *p* < 0.001) than patients who were scheduled for dCRT with CDDP/5FU, no significant differences were seen for other parameters (age, sex, TNM-stage, grading and tumor extension).

**Results:**

Forty-seven patients (25 patients treated with CDDP/5FU and 22 patients treated with Carb/TAX) were evaluated for the intention-to-treat (ITT) analysis and 41 of 47 patients (23 patients treated with CDDP/5FU and 18 patients treated with Carb/TAX) were evaluated for the PP analysis. Severe myelotoxicity (≥ III°) was seen in 52% (CDDP/5FU) and 55% of patients (Carb/TAX), respectively (*p* = 1.000). In the univariate binary logistic regression analysis, patients age was the only factor associated with an increased risk of ≥ III° myelotoxicity (hazard ratio 1.145, 95% CI 1.035; 1.266; *p* = 0.009). Regarding treatment efficiency, no significant differences were seen for overall survival (OS) and freedom from relapse (FFR) between both treatment groups.

**Conclusion:**

Myelotoxicity and oncologic outcome under dCRT were not different for patients with SCC of the esophagus treated with either CDDP/5FU or Carb/TAX. The putative equivalence of dCRT with Carb/TAX in this setting should be further investigated in prospective trials. However, our data reveal that the risk of significant myelotoxicity increases with patient age and therefore other chemotherapy regimens might be evaluated in elderly patients.

## Background

Esophageal cancer is the eighth most common cancer worldwide and in the USA alone almost 17.000 new cases were estimated for 2017 [1, 2]. According to its histology, esophageal cancer is divided into squamous cell carcinoma (SCC) and adenocarcinoma (AC) and epidemiology as well as therapy approaches differ between both subtypes [3, 4].

For patients with locally advanced or node-positive SCCs neoadjuvant chemoradiation therapy (nCRT) with subsequent surgery can improve overall survival (OS) and progression-free survival (PFS) compared with surgery alone [5–7] and therefore has become the treatment of choice for these patients [4, 8]. However, patients who are not suitable for surgery due to medical or technical reasons or patients who refuse surgery should be treated with definite chemoradiation therapy (dCRT).

nCRT is typically done with either cisplatin and 5-fluorouracil (CDDP/5FU) analogous to the older CALGB-trial [6] or with carboplatin and paclitaxel (Carb/TAX) according to the practice-changing CROSS-trial [5]. Although two retrospective analyses found no significant difference for OS and treatment response between both chemotherapy regimens, nCRT with CDDP/5FU was associated with a significantly increased rate of myelotoxicity [9, 10]. In contrast to the neoadjuvant situation, dCRT with Carb/TAX has only been evaluated in some small, retrospective analyses [11–13]. However, both chemotherapy combinations are listed as preferred regimens for dCRT by the National Comprehensive Cancer Network (NCCN) guideline [8]. Honing and colleagues performed a retrospective comparison of CDDP/5FU and Carb/TAX for dCRT in patients with esophageal cancer (EC) [14]. In this study, the authors demonstrated comparable results regarding OS and disease-free survival (DFS) for both chemotherapy regimens, but significantly lower rates of hematologic and non-hematologic toxicity in patients receiving concomitant chemotherapy with Carb/TAX. Importantly, half of the patients in this study were diagnosed with AC which – based on the fundamentally different biology of these neoplasms when compared to SCC- might affect the results, although histology was neither in the univariate nor in multivariate analysis an independent factor for OS or DFS. In addition, the median radiation dose was 50.4 Gray (Gy), which is a relatively low dose. In contrast to the North American guidelines, the German S3-Guideline recommends higher irradiation doses of 50-60Gy for dCRT [4].

At our department, dCRT for SCC patients is routinely administered with Carb/TAX since 2014, while previously treated patients received dCRT with CDDP/5FU. In this study, we compared efficiency and toxicity of dCRT with ≥54Gy and either CDDP/5FU or Carb/TAX for patients with SCC.

## Methods

### Treatment groups

Since 2011, 47 patients with locally advanced or node-positive SCC and without previous chemotherapy treatment were scheduled for dCRT with CDDP/5FU or Carb/TAX at our department.

In a first step, the intention to treat (ITT) analysis retrospectively compared 22 patients who were scheduled for dCRT with at least 54Gy radiation dose and weekly concomitant chemotherapy with carboplatin (area under the curve 2, (AUC 2)) and paclitaxel (50 mg per square meter of body-surface area) to 25 patients who were scheduled for dCRT with at least 54Gy radiation dose and concomitant chemotherapy with cisplatin (20 mg/m^2^/d, bolus infusion) and 5-fluoruracil (500 mg/m^2^/d, 120 h infusion) on day 1–5 and 29–33. To be included into the per protocol (PP) analysis patients had to have received the complete radiation course with at least 54Gy and at least one complete cycle of CDDP/5FU or at least three complete cycles of Carb/TAX. Therefore, six patients (13%) were excluded from the PP analysis. One patient of the CDDP/5FU group was excluded because treatment was terminated when reaching 7.2Gy due to an esophago-tracheal fistula and in one patient chemotherapy with CDDP/5FU was switched to carboplatin alone after only one day because of medical intolerance. In addition, four patients of the Carb/TAX group were excluded from the PP analysis, because they did not receive at least three complete cycles of chemotherapy.

### Radiotherapy

All patients underwent 3-dimensional treatment planning including computed tomography with a slice thickness of 3 mm in supine position. For the delineation of the gross target volume (GTV), which was defined as the primary tumor and macroscopic lymph node metastases, all available diagnostic information (esophago-gastro-duodenoscopy, endoscopic ultrasound (EUS) and 18-Fludeoxyglucose positron emission tomography with combined computed tomography) were used. To generate the planning target volume, a safety margin (radial 1–2 cm; cranio-caudal 4–5 cm) was added to the GTV. In addition, individual modifications like inclusion of the elective, cervical lymphatic pathways were done based on the individual expertise of the treating radiation oncologist. After homogenous irradiation of this volume up to a dose of 41.4–50.4Gy, a local dose escalation was applied to the extended GTV (safety margin of 1–2 cm). While 21 patients (84%) in the CDDP/5FU arm were treated with volumetric modulated arc therapy (VMAT) and 4 patients (16%) were treated with 3-dimensional conformal radiotherapy (3D-CRT), all patients who received Carb/TAX were treated with VMAT using 6−/ or 15 MeV photons. VMAT was performed with a median of two arcs [range 1–3] and 3D-CRT was done with a median of 6 beams [range 5–7]. Median total radiation dose was 54Gy (iqr 54–59.4Gy) with daily doses of 1.8Gy (iqr 1.8–1.8Gy).

#### Patient and tumor baseline characteristics

Table 1 presents patient and tumor characteristics of the ITT and the PP analysis. The median age of patients treated with CDDP/5FU and Carb/TAX was 66 years and 68 years, respectively. 84% of patients in the CDDP/5FU and 68% of patients in the Carb/TAX group were male. The most common T-stage was uT3, which was present in 72% (CDDP/5FU) and 73% (Carb/TAX) of patients. More than 90% of patients in both groups had lymph node metastases. While none of the patients who were scheduled for simultaneous chemotherapy with Carb/TAX had distant metastases, one of the patients scheduled for CDDP/5FU had a supraclavicular lymph node metastasis that was classified as M1 according to the 7th edition of the Classification of Malignant tumours. Since this metastasis was irradiated with the full dose of 54Gy, however, we decided to keep the patient in the analysis. Median primary cranio-caudal tumor extension was 5 cm for both patient groups and all patients had moderately (G2, 56%) or poorly (G3, 44%) differentiated SCCs. Tumor grading was unknown in 4 patients with external histology and endoscopic tumor extension was unknown in 5 patients. No significant differences were seen for any baseline characteristics (age, sex, TNM-stage, tumor grading and tumor extension) between both treatment groups. While there was also no significant difference for the daily radiation dose (median daily radiation dose was 1.8Gy for both groups), patients who were treated with Carb/TAX received a higher total radiation dose than patients treated with CDDP/5FU (median total radiation dose 59.4 Gy vs. 54.0 Gy, *p* < 0.001).

**Table 1:** Patients' and tumor characteristics

Parameter	Intention to treat analysis			Per protocol analysis		
	*Carb/TAX* *n* = 22	CDDP/5FU *n* = 25	*p*-value	*Carb/TAX* *n* = 18	CDDP/5FU *n* = 23	*p*-value
Median Age (IQR)	68 (62–74)	66 (62–69)	0.149	68 (62–72)	66 (58–69)	0.337
Male	15 (68%)	21 (84%)	0.303	13 (72%)	19 (83%)	0.471
T-stage			0.247			0.265
uT1	1 (5%)	0 (0%)		1 (6%)	0 (0%)	
uT2	4 (18%)	2 (8%)		4 (22%)	2 (9%)	
uT3	16 (73%)	27 (72%)		12 (67%)	17 (74%)	
uT4	1 (5%)	5 (20%)		1 (6%)	4 (17%)	
uN+	21 (95%)	23 (92%)	1.000	17 (94%)	21 (91%)	1.000
cM0	22 (100%)	24 (96%)	1.000	18 (100%)	22 (96%)	1.000
Grading			0.756			0.515
G2	11 (61%)	13 (52%)		9 (64%)	12 (52%)	
G3	7 (39%)	12 (48%)		5 (36%)	11 (48%)	
Median tumor extension (cm) (IQR)	5 (3–7)	5 (5–7)	0.216	5 (3–6)	5 (5–6)	0.108
Median radiation dose (Gy) (IQR)	59.4 (55.8–59.4)	54.0 (54.0–54.0)	< 0.001	59.4 (54.0–59.4)	54.0 (54.0–54.0)	< 0.001
Median daily radiation dose (Gy) (IQR)	1.8 (1.8–1.8)	1.8 (1.8–1.8)	0.253	1.8 (1.8–1.8)	1.8 (1.8–2.0)	0.429

*5-FU* 5-fluoruracil, *IQR* inter-quartiles-range, *Gy* gray

### Follow- up

Periodic follow-up examinations with clinical examination, esophago-gastro-duodenoscopy, and computed tomography were done every 3 months, starting approximately 6 weeks after end of dCRT. Local tumor recurrence or lymphnode- and distant metastasis was judged based on all available follow-up data. For the analysis of local tumor control, persistent tumor with positive histology at the time of the first follow-up was classified as local treatment failure.

### Toxicity

Acute myelotoxicity was retrospectively reviewed using medical records and classified according to the Common Terminology Criteria for Adverse Events (CTCAE) v.4.03.

### Statistics

Freedom from relapse (FFR) was calculated for all patients who completed treatment. The respective time interval was defined from the last day of treatment until tumor progression or tumor recurrence. Overall survival (OS) and FFR were calculated from the end of treatment. Statistical analyses comprised comparison of baseline parameters, myelotoxicity and different dose parameters using the Wilcoxon–Mann–Whitney U test or Fishers exact test. OS and FFR where compared using the log-rank test. To further evaluate the influence of baseline characteristics on the risk of ≥ III° myelotoxicity, we also performed a binary logistic regression analysis. All statistical tests were conducted in an exploratory manner on two-sided 5% significance levels using the software *SPSS Statistics 18 version 18.0.0* (IBM SPSS Statistics, Armonk, U. S.).

## Results

### Treatment tolerance

Overall, treatment was well tolerated despite patients’ age and large tumor extension with corresponding large treatment volumes. In the univariate binary logistic regression analysis, patient age was the only factor associated with an increased risk of ≥ III° myelotoxicity (hazard ratio 1.145, 95% CI 1.035; 1.266; *p* = 0.009). In contrast, tumor extension and sex were not associated with a higher risk for ≥ III° myelotoxicity.

No significant differences were seen for the rate of leukopenia, thrombocytopenia, anemia or the cumulative rate of ≥ III° myelotoxicity between both treatment groups (Table 2). In total, ≥ III° myelotoxicity was seen in 52% (CDDP/5FU) and 55% (Carb/TAX) of patients in the ITT-population and in 52% (CDDP/5FU) and 44% (Carb/TAX) of patients in the PP-population. The most common ≥ III° myelotoxicity was leukopenia. In detail, leukopenia I°, II°, III° and IV° was observed 12, 28, 40 and 8% of patients who were scheduled for CDDP/5FU and in 9% (I°), 41% (II°) and 45% (III°) of patients who were scheduled for Carb/TAX. Among patients treated per protocol, leukopenia I°, II°, III° and IV° was seen in 13, 30, 39 and 9% (CDDP/5FU) and in 6% (I°), 44% (II°) and 44% (III°) (Carb/TAX) of patients, respectively. Two patients (8%) who were scheduled for CDDP/5FU and one patient (5%) who was scheduled for Carb/TAX had thrombocytopenia ≥ III° while anemia ≥ III° was observed in none of the patients in the CDDP/5FU group and three patients (14%) in the Carb/TAX group.

**Table 2:** Myelotoxicity

Myelotoxicity	Intention to treat			Per protocol		
	*Carb/TAX* *n* = 22	*CDDP/5FU* *n* = 25	*p*-value	*Carb/TAX* *n* = 18	*CDDP/5FU* *n* = 23	*p*-value
≥ III	12 (55%)	13 (52%)	1.000	8 (44%)	12 (52%)	0.756
Leukopenia			0.634			0.719
0°	1 (5%)	3 (12%)		1 (6%)	2 (9%)	
I°	2 (9%)	3 (12%)		1 (6%)	3 (13%)	
II°	9 (41%)	7 (28%)		8 (44%)	7 (30%)	
III°	10 (45%)	10 (40%)		8 (44%)	9 (39%)	
IV°	0 (0%)	2 (8%)		0 (0%)	2 (9%)	
Thrombocytopenia			0.960			0.364
0°	13 (59%)	16 (64%)		12 (67%)	15 (65%)	
I°	5 (23%)	4 (16%)		5 (28%)	3 (13%)	
II°	3 (14%)	3 (12%)		1 (6%)	3 (13%)	
III°	1 (5%)	2 (8%)		0 (0%)	2 (9%)	
Anemia			0.256			0.854
0°	0 (0%)	1 (4%)		0 (0%)	1 (4%)	
I°	11 (50%)	13 (52%)		10 (56%)	12 (52%)	
II°	8 (36%)	11 (44%)		7 (39%)	10 (43%)	
III°	3 (14%)	0 (0%)		1 (6%)	0 (0%)	

### Local and distant tumor control

Based on all available follow-up information including clinical examination, computed tomography and esophago-gastro-duodenoscopy, 15 patients (60%) treated with CDDP/5FU and 9 patients (41%) treated with Carb/TAX had loco-regional or distant treatment failure (*p* = 0.248). Within the PP-population loco-regional or distant treatment failure was observed in 14 patients (61%) treated with CDDP/5FU and 6 patients (33%) treated with Carb/TAX (*p* = 0.118). In addition, no significant difference was seen for the rate of loco-regional or distant treatment failure within the first year after treatment (40% vs. 36%, *p* = 1.000 (ITT) and 39% vs. 28%, *p* = 0.520 (PP)). The rate of loco-regional recurrence within the first year was 36% in patients treated with CDDP/5FU and 32% in patients treated with Carb/TAX (*p* = 1.000), while distant treatment failure within the first year was seen in 8% (CDDP/5FU) and 18% (Carb/TAX) (*p* = 0.398).

In patients with treatment failure, who were scheduled for dCRT with CDDP/5FU, first site of treatment failure was loco-regional in 11 patients (73%) and first site of treatment failure was distant in 4 patients (27%). Compared to that, in patients with treatment failure, who were scheduled for dCRT with Carb/TAX loco-regional recurrence and distant metastasis was the first site of treatment failure in 4 patients (44%) and 4 patients (44%), respectively. In addition, in one patient (11%) loco-regional and distant treatment failure occurred at the same time (*p* = 0.251). For patients treated per protocol, loco-regional recurrence or distant recurrence was the first site of treatment failure in 10 patients (71%) and 4 patients (29%) treated with CDDP/5FU and in 2 patients (33%) and 3 patients (50%) treated with Carb/TAX. In addition, in one patient (17%) treated with Carb/TAX loco-regional and distant recurrence occurred at the same time (*p* = 0.182).

### Survival

**Fig. 1:**
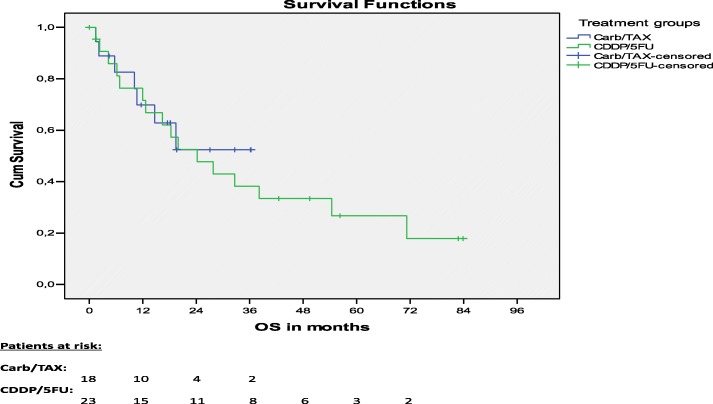
Overall survival

**Fig. 2:**
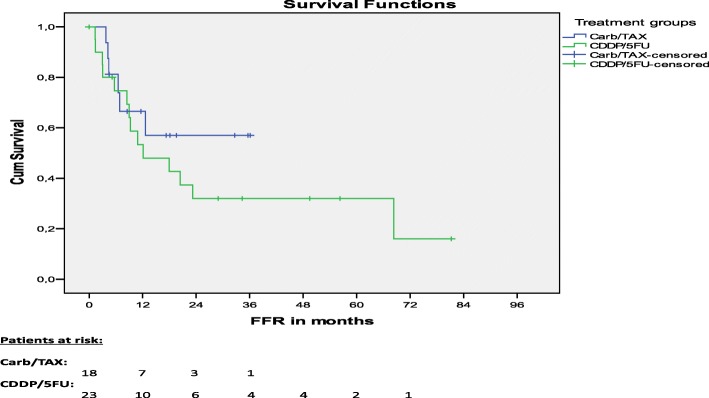
Freedom from relapse

For surviving patients treated per protocol, median follow-up was 23.3 months. Median follow-up was 49.5 months for patients treated with CDDP/5FU and 18.2 months for patients treated with Carb/TAX. While median OS was 24.2 months for patients treated with CDDP/FU, median OS was not reached for patients treated with Carb/TAX. No significant differences were seen for median OS (*p* = 0.784, Fig. 1) and 1-year-OS (72% vs 70%, *p* = 0.902). Median FFR was 12.1 months for patients treated with CDDP/5FU and median FFR was not reached for patients treated with Carb/TAX. No significant differences were seen for median freedom from relapse (FFR) (*p* = 0.359, Fig. 2) and 1-year FFR (53% vs 67%, *p* = 0.524) between both treatment groups.

## Discussion

In this study no significant differences regarding treatment tolerance and oncologic outcome were found between dCRT with either CDDP/5FU or Carb/TAX in patients with squamous cell carcinoma of the esophagus.

While trimodal therapy with nCRT and subsequent surgery has been established as the treatment of choice for SCC patients suitable for surgery, dCRT is recommended for patients unsuitable for surgery due to technical or medical reasons or patients refusal of surgery [4, 8]. Most data in this scenario are derived from studies using concomitant chemotherapy with cisplatin and 5-fluoruracil [15–17]. However, based on the encouraging results of some smaller trials [11–13] and the CROSS-trial [5], which evaluated nCRT with carboplatin and paclitaxel, one might speculate that Carb/TAX might also be used effectively for dCRT.

Our SCC patient cohort revealed no significant differences in OS and FFR between both treatment groups. This result is in line with the results of Honing and colleagues [14], who compared dCRT with CDDP/5FU or Carb/TAX in patients with AC and SCC of the esophagus. While OS of patients treated with CDDP/5FU in our analysis was longer than in the analysis by Honing et al. (median OS 24.2 months vs. 16.1 months), results for patients treated with Carb/TAX are difficult to compare. In our analysis median OS was not reached after a median follow-up time of 18.2 months for surviving patients, while Honing et al. reported a median OS of 13.8 months for patients treated with Carb/TAX. A possible explanation for this difference is the higher rate of patients with distant lymph node metastases (M1a) in Honing’s study, who might have a shorter OS than patients without distant metastases (M0). While distant lymph node metastases were seen in 23% (CDDP/5FU) and 9% (Carb/TAX) of patients in the analysis by Honing et al. [14], in our study only one patient (4%), who was scheduled for CDDP/5FU had distant lymph node metastases. In addition, patients included in the present analysis received higher total radiation doses (median total radiation dose: 54Gy vs. 50.4Gy (CDDP/5FU) and 59.4Gy vs. 50.4Gy (Carb/TAX)). Even this relatively small difference in radiation dose might improve treatment effectiveness and therefore might explain differences in OS. In a retrospective trial by Kim et al. [18], high-dose dCRT (median dose 63Gy) was associated with increased OS and loco-regional control than standard-dose dCRT (median dose 50.4Gy). However, it remains an open question if these results are also applicable to lower total dose differences as seen in the present study.

While OS of patients treated with Carb/TAX in our analysis was also higher than in two other studies evaluating dCRT with Carb/TAX in EC patients [12, 13], it was comparable to the results by Ruppert et al. [11]. In their analysis, patients with locally advanced EC, treated with dCRT and Carb/TAX had a 3-year OS of 56.1%. However, in this study patients also received two cycles of additional chemotherapy after dCRT. Regarding patients treated with CDDP/5FU median OS in our study was longer than in the analyses by Herskovic et al. [15] (12.5 months) and Minsky et al. [17] (18.1 months). But in contrast to our study, both analyses used former (2D and 3D) radiation techniques and a lower radiation dose of 50.4Gy.

Regarding freedom from relapse (FFR), we found no other study analyzing FFR in EC patients treated with dCRT with CDDP/5FU. However, when comparing FFR to DFS and PFS our results for patients receiving CDDP/5FU seem to be comparable to the results of Honing et al. [14] (median FFR of 12.1 months vs. median DFS of 11.1 months). Analogous to the results for OS, results for FFR in our trial are different to compare with other trials because median FFR was not reached. However, 2y-FFR in our trial (57%) is comparable to the results by Ruppert et al. [11] (2y-overall FFR 51.3%). In line with the results by Honing and colleagues [14] no significant difference was seen between both treatment groups.

A total of 48 and 20% of patients treated with CDDP/5FU had loco-regional or distant treatment failure. This is in line with data from Herskovic et al. [15]. In their analysis, patients with EC were treated with 50.4Gy and concomitant chemotherapy with CDDP/5FU. After 12 months loco-regional and distant tumor recurrence was seen in approximately 40 and 22% of patients. In our patients treated with Carb/TAX loco-regional or distant treatment failure was seen in 36 and 27% of patients, respectively. In contrast to that two other trials investigating dCRT with Carb/TAX revealed loco-regional recurrence in 42% of patients [12, 13]. However, in both studies median follow-up was longer than in our study, which might explain the higher rate of loco-regional recurrences. This difference in the median follow-up might also explain the difference in the rate of distant recurrences between our study (27%) and the analysis by Haj Mohammad et al. [12] (42%), in which EC patients were treated with dCRT with Carb/TAX.

In contrast to the results of Honing and colleagues [14] our data revealed no significant differences in terms of myelotoxicity between both treatment groups. Compared to the results presented by Honing et al., the rate of ≥ III° myelotoxicity in our study was higher for both, patients treated with CDDP/5FU (52% vs. 19%) and patients treated with Carb/TAX (55% vs. 4%). This difference is remarkable, because especially for patients treated with Carb/TAX the only noticeable difference is seen for radiation dose (median dose 50.4Gy vs. 59.4Gy). It seems likely, that in this case not only the planning target volume, but also the bone marrow will receive higher doses, which might increase the risk of myelotoxicity. In a study by Noronha et al. [13] ≥ III° leukopenia was seen in 49% of patients treated with dCRT with a median radiation dose of 58.7Gy, which is comparable to our results, but we have to point out that almost 42% of patients in their study received induction chemotherapy. Although the rate of ≥ III° myelotoxicity for patients treated with CDDP/5FU in our analysis is much higher than in the study by Honing et al., it is comparable to other studies. After dCRT with 50.4Gy and concomitant chemotherapy with CDDP/5FU severe or worse myelotoxicity was seen in 48% of patients in a study by Herskovic and colleagues [15]. In addition, ≥ III° myelotoxicity was seen in 58% of patients receiving nCRT with CDDP/5FU [10]. While radiation dose was only 45Gy in this analysis, patients received the same amount of chemotherapy. An overview of different studies evaluating overall survival, rate of recurrence and myelotoxicity of different dCRT regimens for SCC is shown in Table 3.

**Table 3:** Overview of studies evaluating different dCRT regimens for EC

Author (year)	Number of patients	Study design	Histology	Radiotherapy	Simultaneous Chemotherapy	Median overall survival	Local failure	Distant failure	≥ III° heamtologic toxicity
Ruppert, BN (2010) [11]	19	Retrospective	42% SCC	50.4–61.2 Gy; daily dose 1.8–2 Gy	Paclitaxel Carboplatin	1-year OS 68.4%	42.1%	10.5%	46.7% (neutropenia)
Haj Mohammad, N (2014) [12]	127	Retrospective	36% SCC	50.4 Gy (28 Fx.)	Paclitaxel Carboplatin	17.1 months (inoperable patients) 17.4 months (irresectable tumors)	42%	44%	7% (leukopenia)
Noronha, V (2016) [13]	179	Retrospective	92.2% SCC	Mean dose 58.7 Gy in 32 Fx.	Paclitaxel Carboplatin	19 months (1-year OS: 70%)	32%	15%	49% (leukopenia)
Honing, J (2014) [14]	102	Retrospective	50% SCC	Median dose 50.4 Gy, daily dose 1.8–2 Gy	1. Cisplatin 5-FU 2. Paclitaxel Carboplatin	1. 16.1 months 2. 13.8 months	–	–	1. 19% 2. 4%
Herskovic, A (1992) [15]	61 (combined treatment)	Phase 3	84% SCC	50 Gy (25 Fx)	Cisplatin 5-FU	12.5 months (1-year OS: 50%)	43%	22% (after one year)	48%
Minsky, BD (2002) [17]	218	Phase 3	86% SCC	1. 50.4 Gy (28 Fx) 2. 64.8 Gy (36 Fx)	Cisplatin 5-FU	1. 18.1 months 2. 13 months	1. 55% 2. 50%	1. 16% 2. 9%	–
This study	47	Retrospective	100% SCC	1. Median dose 54 Gy (30 Fx.) 2. Median dose 59.4 Gy (33 Fx.)	1. Cisplatin 5-FU 2. Paclitaxel Carboplatin	1. 24.2 months (1-year OS: 72%) 2. Not reached after 18.2 months) (1-year OS 70%)	1. 48% (36% after one year) 2. 36% (32% after one year)	1. 20% (8% after one year) 2. 27% (18% after one year)	1. 52% 2. 55%

While 16% of patients who received CDDP/5FU were irradiated using 3D-CRT, all patients who received Carb/TAX were irradiated using VMAT, but this difference was not statistically significant (*p* = 0.112). In two retrospective analyses no significant differences in terms of oncologic outcome were seen between 3D-CRT and VMAT for EC patients undergoing nCRT or dCRT [19, 20]. However, there was a higher rate of leukopenia in patients undergoing nCRT with VMAT compared to 3D-CRT [19]. Considering the lower radiation dose (45Gy) it remains unclear if this result can be transferred to patients undergoing dCRT.

While the question of our study was similar to the study published by Honing et al. [14], with both studies comparing dCRT with either CDDP/5FU or Carb/TAX in patients with esophageal cancer, there are also some relevant differences. In contrast to our study, Honing and colleagues included patients with different histologic subtypes (adenocarcinoma and squamous cell carcinoma). This has to be mentioned, because chemoradiation is more effective in patients with squamous cell carcinoma than in patients with adenocarcinoma. In addition, patients included in the analysis by Honing et al. were treated with a lower radiation dose of 50.4Gy. Because the German S3-Guideline [4] recommends higher radiation doses of up to 60Gy, results presented by Honing and colleagues probably don’t correspond to the treatment regimens used in the daily routine in Germany.

By only including patients with squamous cell carcinoma of the esophagus, our study can exclude that oncologic outcome is biased by different histology subtypes. In addition, results of our study demonstrate, that the therapeutic equivalence of both chemotherapy regimens can be presumed for treatment concepts with higher total radiation doses.

Because of its retrospective nature this study has some limitations. The most obvious problem is the small number of patients, which clearly compromises the power of the study. On the other side, the small number of patients is also caused be the fact, that we specifically compared dCRT with CDDP/5FU or Carb/TAX only in patients with SCC, which clearly is the biologically more rational approach. A further limitation is the difference regarding the follow-up time. Follow-up of patients treated with CDDP/5FU is clearly longer than follow-up of patients treated with Carb/TAX. The reason for this imbalance is that all patients who received CDDP/5FU were treated between 2011 and 2014 while the first patient who received Carb/TAX was treated at the end of 2014. We have to keep in mind that this difference in follow-up time might contribute to differences in survival and rate of recurrences. To consider the short follow-up of patients treated with Carb/TAX, we also compared one-year OS, one-year FFR and rate of recurrences within one year between both treatment groups and found no significant differences. The last limitation is the difference in the total radiation dose. While patients in the CDDP/5FU group received a median radiation dose of 54Gy, patients in the Carb/TAX group received a median radiation dose of 59.4Gy. As explained before this dose difference might also lead to an increased tumor control probability in patients treated with Carb/TAX. In addition, we cannot rule out that even this dose difference might also impair myelotoxicity. However, both doses are within the recommended dose range for dCRT.

## Conclusion

Myelotoxicity and oncologic outcome under dCRT were not different for patients with SCC of the esophagus treated with either CDDP/5FU or Carb/TAX. The putative equivalence of dCRT with Carb/TAX in this setting should be further investigated in prospective trials. However, our data reveal that the risk of significant myelotoxicity increases with patient age and therefore other chemotherapy regimens might be evaluated in elderly.

## Abbreviations

3D-CRT: 3-dimensional conformal radiotherapy
AC: Adenocarcinoma
Carb/TAX: Carboplatin and paclitaxel
CDDP/5FU: Cisplatin and 5-fluoruracil
dCRT: Definite chemoradiation therapy
DFS: Disease-free survival
EC: Esophageal cancer
FFR: Freedom from relapse
GTV: Gross target volume
Gy: Gray
ITT: Intention-to-treat
NCCN: National Comprehensive Cancer Network
nCRT: Neoadjuvant chemoradiation therapy
OS: Overall survival
PFS: Progression-free survival
PP: Per protocol
SCC: Squamous cell carcinoma
VMAT: Volumetric modulated arc therapy
